# Health Care Workers in the setting of the “Arab Spring”: a scoping review for the Lancet-AUB Commission on Syria

**DOI:** 10.7189/jogh.09.010402

**Published:** 2019-06

**Authors:** Lama Bou-Karroum, Karim N Daou, Mohamed Nomier, Nour El Arnaout, Fouad M Fouad, Fadi El-Jardali, Elie A Akl

**Affiliations:** 1 Center for Systematic Reviews for Health Policy and Systems Research (SPARK), American University of Beirut, Beirut, Lebanon; 2 Department of Health Management and Policy, Faculty of Health Sciences, American University of Beirut, Beirut, Lebanon; 3 Department of Epidemiology and Population Health, Faculty of Health Sciences, American University of Beirut, Beirut, Lebanon; 4 Refugee Health Program, Global Health Institute, American University of Beirut, Beirut, Lebanon; 5 Department of Clinical Epidemiology and Biostatistics, McMaster University, Hamilton, Ontario, Canada; 6 Department of Internal Medicine, Faculty of Medicine, American University of Beirut, Beirut, Lebanon

## Abstract

**Background:**

“Health Care Workers in Conflict Areas” emerged as one of the priority themes for a Lancet Commission addressing health in conflict. The objective of our study was to conduct a scoping review on health workers in the setting of the Syrian conflict, addressing four topics of interest: violence against health care workers, education, practicing in conflict setting, and migration.

**Methods:**

Considering the likelihood of scarcity of data, we broadened the scope of the scoping review to include indirect evidence on health care workers from other countries affected by the “Arab Spring”. We electronically searched six electronic databases. We conducted descriptive analysis of the general characteristics of the included papers. We also used the results of this scoping review to build an evidence gap map.

**Results:**

Out of the 11 165 identified citations, 136 met our eligibility criteria. The majority of the articles tackled the issue of violence against health care workers (63%) followed by practicing in conflict setting (19%), migration (17%) and education (10%). Countries in focus of most articles were: Syria (35%), Iraq (33%), and Bahrain (29%). News, editorials, commentaries and opinion pieces made up 81% of all included papers, while primary studies made up only 9%. All the primary studies identified in this review were conducted on Iraq. Most of the articles about violence against health care workers were on Bahrain, followed by Syria and Iraq. The first and corresponding authors were most frequently affiliated with institutions from non-Arab countries (79% and 79% respectively).

**Conclusions:**

Research evidence on health care workers in the setting of the “Arab Spring” is scarce. This review and the gap map can inform the research agendas of funders and researchers working in the field of health care workers in conflict setting. More well-designed primary studies are needed to inform the decisions of policymakers and other interested parties.

## The Lancet-AUB Commission on Syria

The Lancet and the American University of Beirut (AUB) have launched the ‘Commission on Syria: Health in Conflict’ to raise the profile of the Syrian crisis in global health. The Commission also aims to provide concrete and evidence-informed recommendations addressing the health needs in Syria. Commissioners include thinkers, academics, researchers, and practitioners from multidisciplinary backgrounds. The first meeting of the Commission held in Beirut, Lebanon, in December 2016 convened commissioners, experts, stakeholders, faculty, students, and the Lancet leadership to define the scope of the work. Given the direct attacks on health care workers and the responsibilities of the international community to protect health care workers, “Health Care Workers in Conflict Areas” emerged as one of the priority themes [1,2].

The Commission invited the Center for Systematic Reviews in Health Policy and Systems Research (SPARK) to conduct a scoping review to identify and map the published evidence to support a policy paper addressing the status of health care workers from Syria. The policy paper has since been published under the title “*Health professionals and the weaponisation of healthcare in Syria: A preliminary inquiry for the Lancet-AUB Commission on Syria*” [3].

## The Syrian crisis

On March 2011, anti-government demonstrations began in Syria as part of what has been labeled as the “Arab Spring”. The civil war in Syria that started as merely peaceful demonstrations amid the regional Arab Uprising, has inflicted devastating losses on all levels and sectors including health [4,5]. According to the World Health Organization (WHO), Syria’s civil war has resulted in more than 250 000 deaths and displaced more than 4.8 millions [6]. Syria’s war has created the worst humanitarian and refugee crisis of our time [7,8].

Throughout the crisis, medical facilities and health care workers were being directly and deliberately targeted [9-11]. Physicians for Humans Rights has reported that, since the start of the conflict till September 2016, 782 medical personnel have been killed [9] and an inestimable number of medical professionals were threatened and detained. For example, Syrian authorities criminalized in 2012 the provision of health care to opposition fighters or supporters [12,13]. This contravened international humanitarian law rule that under no circumstances shall any person be punished for carrying out medical activities compatible with medical ethics, regardless of the person benefiting therefrom [13].

The attack on medical facilities and health workers and the lack of security led to an exodus of skilled health professionals [14]. By 2013, 70% of the health workforce had left the country, leaving enormous breach in practice [12,15]. For example, the Syrian American Medical Society (SAMS) reported that in 2014 only 20 doctors, including 2 vascular surgeons and one plastic surgeon, were remaining in Aleppo [16]. The shortage of medical professionals and the increased burden of war injuries led to worsening the quality of health care provided. In addition, medical students and pharmacists who are not fully qualified to provide health care had to provide care in absence of trained professionals [16]. All these traumatizing experiences subjected the medical personnel to psychological trauma, depression and burn out [15].

The objective of our study was to conduct a scoping review on health care workers in the setting of the Syrian conflict, addressing four topics of interest: violence against health care workers, education, practicing in conflict setting, and migration. Considering the likelihood of scarcity of data, we broadened the scope of the scoping review to include indirect evidence on health care workers from other countries affected by the “Arab Spring”.

## METHODS

### Scoping review method

We conducted a scoping review, which is typically used to present “a broad overview of the evidence pertaining to a topic, irrespective of study quality, to examine areas that are emerging, to clarify key concepts and to identify gaps” [17]. We followed Joanna Briggs Institute (JBI) guidelines for conducting and reporting scoping reviews [17]. Similarly, and for developing the evidence gap map, we relied on the results of a methodological review on these maps [18].

### Protocol

We developed a protocol for this scoping review following the PROSPERO format, which is available upon request from the corresponding author.

### Eligibility criteria

#### Population and setting of interest

Our main scope of interest consisted of health care workers from Syria affected by the ongoing Syrian conflict. As mentioned above, we broadened the scope to include indirect evidence on health care workers from other countries affected by the “Arab Spring”, as detailed below.

- Health care workers of interest included: midwives, nurses, paramedics, pharmacists, physicians, laboratory technicians, as well as medical students and trainees. We focused on these types of health care workers given their prominent roles in conflict settings.
- The Arab spring refers to the series of protests and demonstrations and subsequent violent events across Arab countries that commenced in December 2010. These countries include Bahrain, Egypt, Libya, Syria, Tunisia, and Yemen. We also included Iraq given the similarities to the Syrian context in terms the autocratic aspect of the regime and the rise of Islamic State in Iraq and Syria (ISIS). Some analysts argue that the Iraqi conflict is tightly related to the Arab Spring [19]. We did not include other countries such as Morocco and Algeria where only demonstrations took place without major insurgencies, civil uprisings and violence.
- Studies about health care workers that relate to one of the above countries of interest, but in a different setting (e.g., European countries) were also eligible.
- The topic of health care workers in conflict setting should have been addressed in at least the title, abstract or a full paragraph of the paper in order for the study to be eligible for inclusion.

#### Study design

We included all types of study designs, including: news, editorials, commentaries, opinion pieces, technical reports, primary studies, narrative reviews, systematic reviews, and policy briefs. We restricted our eligibility criteria to articles published in peer-reviewed journals.

### Literature search

We searched the following electronic databases: MEDLINE, PubMed, EMBASE, Human Resources for Health (HRH) Global Resource Center, the World Health Organization (WHO) global Health Library and the Cochrane Central Register of Controlled Trials (CENTRAL). We ran for each of these databases separate searches for each of the countries of interest starting from the date that country was affected by the conflict, and up to January 12, 2017 (February 2, 2017 for CENTRAL).

We developed the search strategies with the help of an experienced librarian (see Appendix S1 in **Online Supplementary Document** for details of the search strategies). They included both index terms and free text words for the two following concepts: health care workers and the country of interest. When running the search, we ANDed the terms for these two concepts. We did not limit to specific languages, but limited to human studies. We also screened the reference lists of included studies.

### Selection process

In a first step, teams of two reviewers used the above eligibility criteria to screen titles and abstracts of identified citations for potential eligibility independently and in duplicate. We obtained the full text for citations judged as potentially eligible by at least one of the two reviewers. Next, teams of two reviewers screened in duplicate and independently the full texts for eligibility using standardized and pilot tested screening forms. They resolved disagreements by discussion and when needed with the help of a third reviewer. We conducted calibration exercises to ensure the validity of the selection process.

### Data abstraction

One reviewer abstracted data using standardized and pilot tested forms and another reviewer verified the abstracted data. Any disagreement between the initial abstractor and the verifier was resolved by discussion and when needed with the help of a third reviewer. We conducted calibration exercises to ensure the validity of the data abstraction process.

We abstracted from each paper the following information:

- Last name of first author
- Year of publication
- Type of publication (e.g., news, editorial, primary study, systematic review)
- Language of publication
- Country of affiliation of the corresponding author
- Country of affiliation of first author
- Characteristics of the journal of publication (name and impact factor (impact factor was extracted for the year the search was performed).
- Country(ies) from which the HCW subject of the paper come
- Year the study was conducted
- Sample size
- Theme(s) of the study:
  1. Violence against health care workers: refers to attacks and violent acts against health care workers such as killing, arrest and kidnapping.
  2. Education: refers to training and education (e.g. continuing medical education) received by professionals in conflict zones.
  3. Practicing in conflict setting: refers to the special practices (e.g. clinical) and conditions under which health workers practice in conflict setting.
  4. Migration: refers to the issue of migration, movement and exodus of health care workers from conflict zones.
  5. Other
- Key findings that relate to the above themes

### Data synthesis

We conducted descriptive analysis of the general characteristics of the included papers. We graphically represented the annual production of papers by country. We presented a narrative summary of the key findings of the included studies categorized by themes (violence, migration, education and practice in conflict setting). We also used the results of this scoping review to build an evidence gap map. Evidence maps are defined as “a systematic search of a broad field to identify gaps in knowledge and/or future research needs”. These maps present results in a user-friendly format, often a visual figure or graph, cross-tables or a searchable database [18]. We planned to present the gaps by country, study design and topic.

## RESULTS

Out of the 11 165 citations identified, 136 met our eligibility criteria. Full list of included studies is provided in Appendix S2 in **Online Supplementary Document**. The folder is organized by topic and each file is named in a way to indicate the country, the last name of the first author, the year, and the full title of the paper.

**Figure 1:**
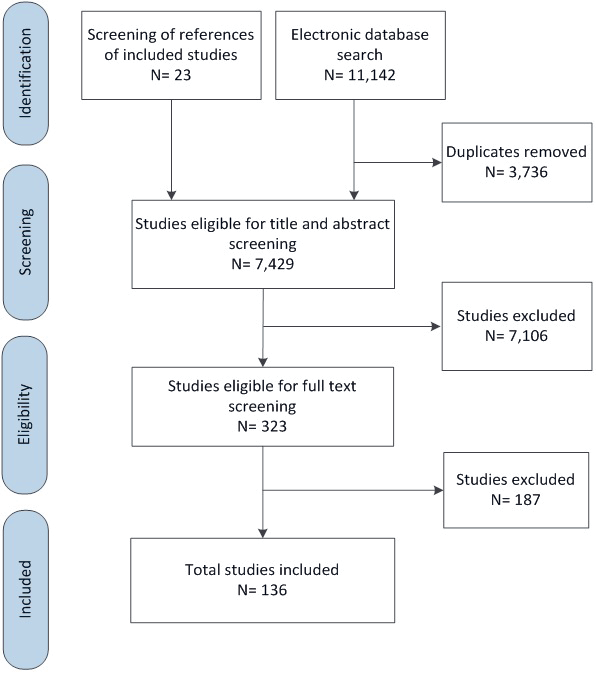
PRISMA flowchart.

**Figure 1** summarizes the selection process. At the full text screening stage, we excluded 187 papers for the following reasons: not about health care workers (n = 82), not country of interest (n = 64), not conflict situation (n = 37), not published in peer-reviewed journals (n = 4).

### Characteristics of the included papers

**Table 1** presents the characteristics of the included papers. The majority of the articles tackled the issue of violence against health care workers (63%). The first and corresponding authors were affiliated with institutions from non-Arab countries (79% and 79% respectively), mainly United Kingdom (UK) and United States (US). The top countries focus of the articles were Syria (35%), Iraq (33%) and Bahrain (29%). We identified no articles on Tunisia. News, editorials, commentaries and opinion pieces made up 81% of all included papers. Primary studies made up only 9% of the papers. The majority of articles (95%) were published in English.

**Table 1:** General characteristics of the included papers (N = 136)

Characteristics of the included papers	n (%)
Countries of Health Care Workers:*	
Syria	47 (35%)
Iraq	45 (33%)
Bahrain	39 (29%)
Egypt	3 (2%)
Libya	3 (2%)
Yemen	1 (1%)
Tunisia	0 (0%)
**Subjects of the papers:†**	
Violence against health workers	86 (63%)
Practising in conflict setting	26 (19%)
Migration	23 (17%)
Education	14 (10%)
**Country of the institution to which the first author is affiliated: ‡**	
Arab countries	7 (5%)
Non-Arab countries	107 (79%)
-United Kingdom	54 (40%)
-United States	31 (23%)
-Other	22 (16%)
**Country of the institution to which the corresponding author is affiliated:‡**	
Arab countries	6 (4%)
Non-Arab countries:	108 (79%)
-United Kingdom	52 (38%)
-United States	30 (22%)
-Other	26 (19%)
**Type of publication:**	
News	57 (42%)
Editorials; commentaries; opinion pieces	53 (39%)
Primary studies	12 (9%)
Case studies	7 (5%)
Letter to editor; correspondence	5 (4%)
Technical reports	1 (1%)
Narrative Review	1 (1%)
Systematic Review	0 (0%)
Language of publication:	
English	129 (95%)
German	6 (4%)
Dutch	1 (1%)

*Two papers addressed both Syria and Bahrain.

†One paper may have more than one option that applies.

‡22 papers did not report on the names and affiliations of authors.

**Figure 2:**
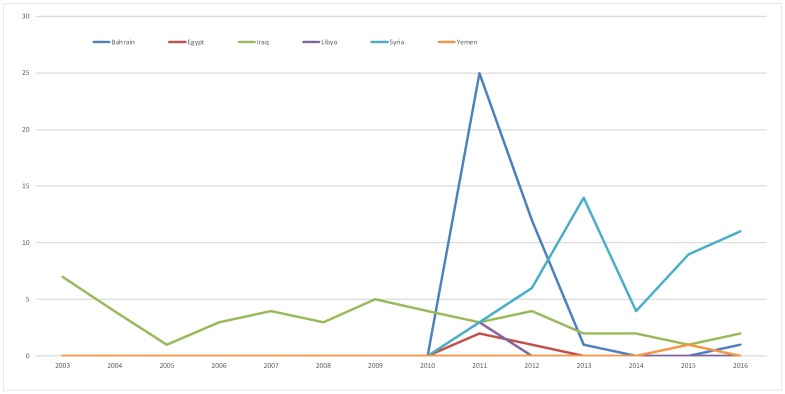
Annual production of evidence on Health Care Workers (HCWs) in Syria and other “Arab Spring” countries.

As shown in **Figure 2**, most articles were published in 2011 (N = 36). The publication of papers on Bahrain peaked in 2011 then decreased after 2012. For papers on Syria, the peak of publication was in 2013 then decreased to re-emerge in 2016. The trend of publication of papers on Iraq did not show major fluctuations over the years.

### Characteristics of the journals

The articles were published across 39 journals. The top journals in which the included papers were published were the BMJ (33.3%), the Lancet (25.0%), Nursing Standard (5.9%), JAMA (3.7%), Deutsche Apotheker Zeitung (3.7%) and CMAJ (3%). The median impact factor of the 39 journals publishing the included articles was 19.967 (interquartile range = 19.967- 44.002).

### Evidence Gap Map

**Figure 3:**
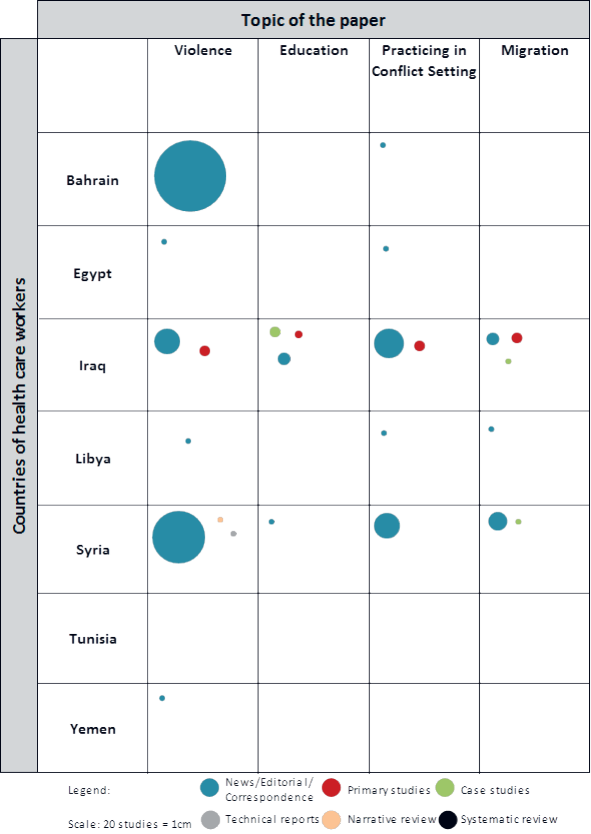
Evidence gap map related to health care workers from Syria and other “Arab Spring” countries.

**Figure 3** presents the evidence gap map about health care from Syria and other “Arab Spring” Countries. It provides a visual overview of existing evidence across countries, study designs and subjects. The gap map shows that the primary studies identified in this review were conducted on Iraq across all the topics (**Figure 3**). Most of the articles about violence against health care workers were on Bahrain, followed by Syria and Iraq. All the articles about violence in Bahrain and the majority of the articles about violence in Syria were news, editorials or correspondence articles.

### Key findings

We organized the key findings of the included papers by themes (education, migration, practice in conflict setting and violence). **Table 2** presents the key findings of included studies. We presented the key findings narratively below:

**Table 2:** Key findings of included studies

Theme	Key findings
Education	Negative consequence of conflict on education include: inadequate competencies, lower quality of education, limited professional development.
	Main training areas needed in conflict setting: emergency medicine, trauma care, first aid and midwifery.
	Main providers of training: international aid organizations.
Migration	Migration consequences: shortage of health workers, weakening the health system, raising serious challenges to future reconstruction effort.
	Factors predicting migration from conflict areas: financial issues, security and training concerns
Practicing in conflict setting	Practice settings in conflict suffer from: shortages of water, electricity and poor sanitation, lack of equipment, supplies and drugs.
	Heath workers practicing in conflict settings lack expertise mainly in emergency medicine.
Violence	Health care workers in conflict setting being arrested, tortured, and killed contravening humanitarian law.
	Health workers were also threatened by the different armed groups to force them to treat their members preferentially breaching medical neutrality.
	Doctors were getting assaulted, violated, and humiliated (in emergency departments) due to lack of security, internal political corruption, and inadequate repartition of physician.

#### Education

All 14 articles addressing the issue of education of HCWs were from Iraq except one from Syria. These studies found that conflict negatively affect the education and training of health workers leading to inadequate competencies, lower quality of education and limited professional development [20-23]. The training areas commonly tackled by the papers were emergency medicine, trauma care, first aid and midwifery [20,24-29]. The training were mainly provided by international aid organizations and through distant learning such as tele-education [20,23,28-31].

#### Migration

All 23 articles tackling the issue of migration of HCWs were about Iraq and Syria except for one about Libya. Most articles described how migration led to shortage of health workers in conflict settings thus weakening the health system and raising serious challenges to future reconstruction efforts [32-36]. Factors commonly identified as predicting physicians’ emigration from conflict areas were financial issues, security and training concerns [32,33,37,38].

#### Practicing in conflict settings

The 26 articles on practicing in conflict settings were mostly about Iraq and Syria. They found that health workers in conflict setting are faced with lack of equipment, supplies and drugs [39-41]. In addition, practice settings suffer from shortages of water, electricity and poor sanitation [40-42]. Couple of articles highlighted how most heath workers practicing in conflict settings lack expertise mainly in emergency medicine [25,39,43]. One study found that physicians are using the internet to consult with overseas physicians who offer assistance on non-emergency cases [44].

#### Violence

The vast majority of articles on violence against health care workers (82 out of 86) were about Bahrain, Syria and Iraq. Topics included health care workers being arrested, tortured, and killed, for helping injured protesters; breaching medical neutrality; and contravening humanitarian law.

In Bahrain, papers reported on health workers (including doctors, nurses and paramedics) being charged for treating pro-democracy protestors during the civil and political unrest of 2011 [45-51]. Papers reported on health professionals being systematically abducted, detained, threatened and tortured by the government forces [48,50-57]. There were also reports of health care workers being accused of anti-government activities during political protests in Bahrain having their salaries stopped [58,59], with some of sentenced to up to 15 years in jail [58,60,61].

In Syria, included articles reported direct and systematic attack on medical personnel and medical facilities. Ben Taleb et al described how the deterioration of health care was not only a consequence of the conflict, but in many instances a result of this systematic targeting of hospitals and medical staff [62]. Abbara et al reported that in 2014, one health worker was, on average, killed every other day [12]. Health workers were also threatened by the different armed groups to force them to treat their members preferentially. There were also reports that some doctors were arrested, tortured, and killed, for helping injured protesters; other doctors who support the regime reportedly had to show loyalty by mishandling injured people or letting them die [12,63-66].

In Iraq, Al-Kindi et al reported that doctors were getting assaulted, violated, and humiliated [67]. A study found that 80% of emergency department doctors reported were assaulted by a patient or their family member at least once within the last year; and 38% reported were threatened with a gun [25]. In another study 41% of nurses reported being physically attacked [68]. Investigators hypothesized that these assaults might have resulted from bereavement, lack of security, internal political corruption, and inadequate repartition of physicians [67,68]. There were also reports of Iraqi doctors, academics, and scientists being assassinated and kidnapped, following the 2003 US led invasion of the country. There seemed to be a systematic targeting of the brightest, most distinguished, and most highly regarded doctors and scientists articles [69].

## DISCUSSION

We conducted a scoping review on health care workers in the setting of the Syrian conflict and the “Arab Spring”, addressing four topics of interest: violence against health care workers, education, practicing in conflict setting, and migration. The resulting evidence gap map provides an overview of available evidence for the countries of interest, by study design, and by topic of the paper (violence, education, practicing and migration), while specifying the number of studies. It also highlights the gaps by topic and by country.

The majority of included articles were about either Syria, Iraq or Bahrain, and addressed the topic of violence against health care workers. Articles about Bahrain and Syria reported that health care workers were systematically arrested, threatened, tortured and killed by the government forces for helping protesters. Articles about Syria reported direct and systematic targeting of medical facilities and medical personnel. The highest number of reports about violence against health care workers came from Bahrain.

The included articles described how conflict affected the education and training of health workers leading to inadequate competencies, lower quality of education and limited professional development. They also described how migration led to shortage of health workers in conflict settings thus weakening the health system and raising serious challenges to future reconstruction efforts. Factors commonly identified as predicting physicians’ emigration from conflict areas were financial issues, security and training concerns. Challenges to health care workers in conflict settings included the lack of equipment, supplies and drugs in addition to shortages of water and electricity and poor sanitation. A number of articles highlighted how most heath workers practicing in conflict settings lacked expertise mainly in emergency medicine.

The major finding of this scoping review is the scarcity of research evidence. Primary studies made up less than 10% of all papers. The predominance of news, opinion pieces and commentaries (more than 80% of all papers) likely reflects the challenges in conducting research in conflict areas [70]. Patel et al. reported on the challenges to systematically collect data on violence against local health workers in conflict settings. These challenges included security reasons, insufficient research capacity, bias in data collection, insufficient research funding and a lack of developed method [71].

The scarcity of research evidence might also reflect the lack of the local capacity for conducting research. Indeed, most of the identified papers had primary authors affiliated with institutions from Non-Arab countries. Even before the advent of the “Arab Spring”, countries from the region had limited production of health research [72,73]. Researchers from the region blamed this limited production on the limited financial and human resources capacity [74]. At the same time, the relatively high number of publication by researchers from the US and the UK in high impact journals indicate the interest of the international research community as well as journals in the topic of health care workers in conflict setting.

To our knowledge, this is the first study to scope the published evidence about health care workers from Syria and other “Arab Spring” countries. One strength of the study is that we followed the JBI guidelines for conducting scoping reviews [17] and a methods review of the literature to build our gap map [18]. This study has some limitations. First, by not using language restrictions we were able to include studies published in languages other than English. Second, we did not search Scopus and Web of Science, which are two major databases. However, we have searched 6 important and relevant databases for this review so we believe we have captured a large number of relevant studies. The scoping methodology of this paper could be replicated in the future to map out the published evidence on health care workers in conflict setting not restricted to Arab countries. The evidence gap map can inform the agendas of both funders and researchers working in the field of health care workers in conflict settings. Additionally, the process of starting with a scoping review can be replicated for future Lancet Commissions to guide their work and provide them with an overview of the existing evidence and gaps. The findings of this review can also inform the work of the WHO that has developed a new tracking system for documenting and collecting data on attacks on health care workers and health facilities in armed conflicts. The tracking system feeds into a repository for reports from governments, media and civil society organizations [71].

There is a need for more rigorous and well-designed primary studies to inform the decision-making process of policymakers and other interested stakeholders. Researchers should also develop methods and guidelines on conducting and reporting research on health care workers in conflict settings taking into consideration the challenges in this field. Future studies should also assess effective interventions and develop context-specific and evidence-based guidelines to protect health care workers exposed to conflict-related violence. Investigators should consider including process evaluation and qualitative studies to explore the barriers and facilitators for these interventions and to better understand the circumstances and motives of these health care workers in different contexts.

## Additional Material

Online Supplementary Document
