# Supplementary material for: Health Care Workers in the setting of the “Arab Spring”: a scoping review for the Lancet-AUB Commission on Syria
Source: J Glob Health. 2018 Oct 29;9(1):010402. doi: 10.7189/jogh.09.010402 (PMC6207103; doi:10.7189/jogh.09.010402)
Supplement: Online Supplementary Document [file jogh-09-010402-s001.pdf]

Online Supplementary Document

Bou-Karroum et al. Health Care Workers in the setting of the "Arab Spring": a scoping review for the Lancet-AUB Commission on Syria

J Glob Health 2018;8:020408

Appendix S1: Search strategy

EMBASE

Database: Embase <1980 to 2017 Week 02>

Search Strategy:

----

1 exp health care personnel/ (1475167)

2 ((health or healthcare or medical) adj2 (worker* or professional* or personnel or manpower or workforce)).tw. (156278)

3 (doctor* or nurse or nurses or nursing or physician* or midwife or midwives or midwifery or paramedic* or medic or medics or pharmacist*).tw. (970010)

4 (birth adj attendant*).tw. (1736)

5 ((lab or laboratory) adj technician*).tw. (1660)

6 (medical adj (resident or residents or graduate* or student*)).tw. (40861)

7 (human adj resource*).tw. (8565)

8 or/1-7 (1941738)

9 bahrain/ or (bahrain or bahraini or bahrainis).tw. (1560)

10 8 and 9 (319)

11 limit 10 to yr="2011 -Current" (165)

****

Database: Embase <1996 to 2017 January 11>

Search Strategy:

----

1 exp health care personnel/ (1282916)

2 ((health or healthcare or medical) adj2 (worker* or professional* or personnel or manpower or workforce)).tw. (136157)

3 (doctor* or nurse or nurses or nursing or physician* or midwife or midwives or midwifery or paramedic* or medic or medics or pharmacist*).tw. (720303)
4 (birth adj attendant*).tw. (1453)
5 ((lab or laboratory) adj technician*).tw. (1265)
6 (medical adj (resident or residents or graduate* or student*)).tw. (32729)
7 (human adj resource*).tw. (7450)
8 or/1-7 (1556051)
9 egypt/ or (egypt or egyptians or egyptian).tw. (20787)
10 8 and 9 (1877)
11 limit 10 to yr="2011 -Current" (1198)

****
Database: Embase <1980 to 2017 Week 02>
Search Strategy:
----
1 exp health care personnel/ (1475167)
2 ((health or healthcare or medical) adj2 (worker* or professional* or personnel or manpower or workforce)).tw. (156278)
3 (doctor* or nurse or nurses or nursing or physician* or midwife or midwives or midwifery or paramedic* or medic or medics or pharmacist*).tw. (970010)
4 (birth adj attendant*).tw. (1736)
5 ((lab or laboratory) adj technician*).tw. (1660)
6 (medical adj (resident or residents or graduate* or student*)).tw. (40861)
7 (human adj resource*).tw. (8565)
8 or/1-7 (1941738)
9 Iraq/ or (iraq or iraqi or iraqis).tw. (10016)
10 8 and 9 (1477)
11 limit 10 to yr="2003 -Current" (1319)

****
Database: Embase <1980 to 2017 Week 02>
Search Strategy:
----
1 exp health care personnel/ (1475167)
2 ((health or healthcare or medical) adj2 (worker* or professional* or personnel or manpower or workforce)).tw. (156278)

3 (doctor* or nurse or nurses or nursing or physician* or midwife or midwives or midwifery or paramedic* or medic or medics or pharmacist*).tw. (970010)
4 (birth adj attendant*).tw. (1736)
5 ((lab or laboratory) adj technician*).tw. (1660)
6 (medical adj (resident or residents or graduate* or student*)).tw. (40861)
7 (human adj resource*).tw. (8565)
8 or/1-7 (1941738)
9 libya/ or (libya or libyens or libyen).tw. (1712)
10 8 and 9 (221)
11 limit 10 to yr="2011 -Current" (100)

****
Database: Embase <1980 to 2017 Week 02>
Search Strategy:
----
1 exp health care personnel/ (1475167)
2 ((health or healthcare or medical) adj2 (worker* or professional* or personnel or manpower or workforce)).tw. (156278)
3 (doctor* or nurse or nurses or nursing or physician* or midwife or midwives or midwifery or paramedic* or medic or medics or pharmacist*).tw. (970010)
4 (birth adj attendant*).tw. (1736)
5 ((lab or laboratory) adj technician*).tw. (1660)
6 (medical adj (resident or residents or graduate* or student*)).tw. (40861)
7 (human adj resource*).tw. (8565)
8 or/1-7 (1941738)
9 syria/ or (syria or syrians or syrian).tw. (11628)
10 9 not (syrian adj2 hamster*).mp. (2769)
11 8 and 10 (370)
12 limit 11 to yr="2011 -Current" (259)

****
Database: Embase <1980 to 2017 Week 02>
Search Strategy:
----
1 exp health care personnel/ (1475167)
2 ((health or healthcare or medical) adj2 (worker* or professional* or personnel or manpower or workforce)).tw. (156278)

3 (doctor* or nurse or nurses or nursing or physician* or midwife or midwives or midwifery or paramedic* or medic or medics or pharmacist*).tw. (970010)
4 (birth adj attendant*).tw. (1736)
5 ((lab or laboratory) adj technician*).tw. (1660)
6 (medical adj (resident or residents or graduate* or student*)).tw. (40861)
7 (human adj resource*).tw. (8565)
8 or/1-7 (1941738)
9 Tunisia/ or (tunisia or tunisians or tunisian).tw. (11828)
10 8 and 9 (690)
11 limit 10 to yr="2011 -Current" (380)

****

Database: Embase <1980 to 2017 Week 02>

Search Strategy:

----

1 exp health care personnel/ (1475167)
2 ((health or healthcare or medical) adj2 (worker* or professional* or personnel or manpower or workforce)).tw. (156278)
3 (doctor* or nurse or nurses or nursing or physician* or midwife or midwives or midwifery or paramedic* or medic or medics or pharmacist*).tw. (970010)
4 (birth adj attendant*).tw. (1736)
5 ((lab or laboratory) adj technician*).tw. (1660)
6 (medical adj (resident or residents or graduate* or student*)).tw. (40861)
7 (human adj resource*).tw. (8565)
8 or/1-7 (1941738)
9 yemen/ or (yemen or yemenis or yemeni).tw. (2124)
10 8 and 9 (230)
11 limit 10 to yr="2011 -Current" (115)

****

Medline

Database: Epub Ahead of Print, In-Process & Other Non-Indexed Citations, Ovid MEDLINE(R) Daily and Ovid MEDLINE(R) <1946 to Present>
Search Strategy:
----
1 health manpower/ or exp health personnel/ (491066)
2 ((health or healthcare or medical) adj2 (worker* or professional* or personnel or manpower or workforce)).tw. (139545)
3 (doctor* or nurse or nurses or nursing or physician* or midwife or midwives or midwifery or paramedic* or medic or medics or pharmacist*).tw. (886047)
4 (birth adj attendant*).tw. (2078)
5 ((lab or laboratory) adj technician*).tw. (1379)
6 (medical adj (resident or residents or graduate* or student*)).tw. (36844)
7 (human adj resource*).tw. (8192)
8 or/1-7 (1280119)
9 Bahrain/ or (Bahrain or bahraini or Bahrianis).tw. (890)
10 8 and 9 (193)
11 limit 10 to yr="2011 -Current" (89)

****

Database: Epub Ahead of Print, In-Process & Other Non-Indexed Citations, Ovid MEDLINE(R) Daily and Ovid MEDLINE(R) <1946 to Present>
Search Strategy:
----
1 health manpower/ or exp health personnel/ (491066)
2 ((health or healthcare or medical) adj2 (worker* or professional* or personnel or manpower or workforce)).tw. (139545)
3 (doctor* or nurse or nurses or nursing or physician* or midwife or midwives or midwifery or paramedic* or medic or medics or pharmacist*).tw. (886047)
4 (birth adj attendant*).tw. (2078)
5 ((lab or laboratory) adj technician*).tw. (1379)
6 (medical adj (resident or residents or graduate* or student*)).tw. (36844)
7 (human adj resource*).tw. (8192)
8 or/1-7 (1280119)
9 egypt/ or (egypt or egyptians or egyptian).tw. (22084)

10 8 and 9 (1406)

11 limit 10 to yr="2011 -Current" (478)

Database: Epub Ahead of Print, In-Process & Other Non-Indexed Citations, Ovid MEDLINE(R) Daily and Ovid MEDLINE(R) <1946 to Present>

Search Strategy:

----

1 health manpower/ or exp health personnel/ (491066)

2 ((health or healthcare or medical) adj2 (worker* or professional* or personnel or manpower or workforce)).tw. (139545)

3 (doctor* or nurse or nurses or nursing or physician* or midwife or midwives or midwifery or paramedic* or medic or medics or pharmacist*).tw. (886047)

4 (birth adj attendant*).tw. (2078)

5 ((lab or laboratory) adj technician*).tw. (1379)

6 (medical adj (resident or residents or graduate* or student*)).tw. (36844)

7 (human adj resource*).tw. (8192)

8 or/1-7 (1280119)

9 Iraq War, 2003-2011/ or Iraq/ (6913)

10 Iraq/ or (iraq or iraqi or iraqis).mp. (9872)

11 9 or 10 (9872)

12 8 and 11 (1062)

13 limit 12 to yr="2003 -Current" (879)

Database: Epub Ahead of Print, In-Process & Other Non-Indexed Citations, Ovid MEDLINE(R) Daily and Ovid MEDLINE(R) <1946 to Present>

Search Strategy:

----

1 health manpower/ or exp health personnel/ (491066)

2 ((health or healthcare or medical) adj2 (worker* or professional* or personnel or manpower or workforce)).tw. (139545)

3 (doctor* or nurse or nurses or nursing or physician* or midwife or midwives or midwifery or paramedic* or medic or medics or pharmacist*).tw. (886047)

4 (birth adj attendant*).tw. (2078)

5 ((lab or laboratory) adj technician*).tw. (1379)

6 (medical adj (resident or residents or graduate* or student*)).tw. (36844)

7 (human adj resource*).tw. (8192)

8 or/1-7 (1280119)

9 libya/ or (libya or libyan or libyans).tw. (1733)
10 8 and 9 (166)
11 limit 10 to yr="2011 -Current" (53)

****

Database: Epub Ahead of Print, In-Process & Other Non-Indexed Citations, Ovid MEDLINE(R) Daily and Ovid MEDLINE(R) <1946 to Present>

Search Strategy:

----

1 health manpower/ or exp health personnel/ (491066)
2 ((health or healthcare or medical) adj2 (worker* or professional* or personnel or manpower or workforce)).tw. (139545)
3 (doctor* or nurse or nurses or nursing or physician* or midwife or midwives or midwifery or paramedic* or medic or medics or pharmacist*).tw. (886047)
4 (birth adj attendant*).tw. (2078)
5 ((lab or laboratory) adj technician*).tw. (1379)
6 (medical adj (resident or residents or graduate* or student*)).tw. (36844)
7 (human adj resource*).tw. (8192)
8 or/1-7 (1280119)
9 syria/ or (syria or syrians or syrian).mp. (12006)
10 9 not (syrian adj2 hamster*).mp. (2649)
11 8 and 10 (282)
12 limit 11 to yr="2011 -Current" (167)

Database: Epub Ahead of Print, In-Process & Other Non-Indexed Citations, Ovid MEDLINE(R) Daily and Ovid MEDLINE(R) <1946 to Present>

Search Strategy:

----

1 health manpower/ or exp health personnel/ (491066)
2 ((health or healthcare or medical) adj2 (worker* or professional* or personnel or manpower or workforce)).tw. (139545)
3 (doctor* or nurse or nurses or nursing or physician* or midwife or midwives or midwifery or paramedic* or medic or medics or pharmacist*).tw. (886047)
4 (birth adj attendant*).tw. (2078)
5 ((lab or laboratory) adj technician*).tw. (1379)
6 (medical adj (resident or residents or graduate* or student*)).tw. (36844)
7 (human adj resource*).tw. (8192)

8 or/1-7 (1280119)
9 Tunisia/ or (Tunisia or Tunisian or Tunisians).tw. (9953)
10 8 and 9 (401)
11 limit 10 to yr="2011 -Current" (142)

Database: Epub Ahead of Print, In-Process & Other Non-Indexed Citations, Ovid MEDLINE(R) Daily and Ovid MEDLINE(R) <1946 to Present>
Search Strategy:
----
1 health manpower/ or exp health personnel/ (491066)
2 ((health or healthcare or medical) adj2 (worker* or professional* or personnel or manpower or workforce)).tw. (139545)
3 (doctor* or nurse or nurses or nursing or physician* or midwife or midwives or midwifery or paramedic* or medic or medics or pharmacist*).tw. (886047)
4 (birth adj attendant*).tw. (2078)
5 ((lab or laboratory) adj technician*).tw. (1379)
6 (medical adj (resident or residents or graduate* or student*)).tw. (36844)
7 (human adj resource*).tw. (8192)
8 or/1-7 (1280119)
9 Yemen/ or (Yemen or Yemeni or Yemenis).tw. (2005)
10 8 and 9 (168)
11 limit 10 to yr="2011 -Current" (56)

PubMed

Search	Query	Items found
#38	Search #14 AND #36 Filters: Publication date from 2011/01/01 to 2017/12/31	81
#37	Search #14 AND #36	200
#36	Search "bahrain"[MeSH Terms] OR "bahrain"[tw] OR "bahraini" [tw] OR "bahrainis" [tw]	841
#14	Search #3 OR #4 OR #5 OR #6 OR #7 OR #8 OR #10 OR #13	1450762
#13	Search "physicians"[MeSH Terms] OR "physicians"[tw] OR "doctor"[tw] OR ("physicians"[MeSH Terms] OR "physicians"[tw] OR "doctors"[tw]) OR ("nurses"[MeSH Terms] OR "nurses"[tw] OR "nurse"[tw] OR "nursing"[tw]) OR ("nurses"[MeSH Terms] OR "nurses"[tw]) OR ("physicians"[MeSH Terms] OR "physicians"[tw] OR "physician"[tw]) OR ("physicians"[MeSH Terms] OR "physicians"[tw]) OR ("midwifery"[MeSH Terms] OR "midwifery"[tw] OR "midwife"[tw]) OR ("midwifery"[MeSH Terms] OR "midwifery"[tw] OR "midwives"[tw]) OR ("midwifery"[MeSH Terms] OR "midwifery"[tw]) OR ("emergency medical technicians"[MeSH Terms] OR ("emergency"[tw] AND "medical"[tw] AND "technicians"[tw]) OR "emergency medical technicians"[tw] OR "paramedic"[tw] OR "allied health personnel"[MeSH Terms] OR ("allied"[tw] AND "health"[tw] AND "personnel"[tw]) OR "allied health personnel"[tw]) OR ("allied health personnel"[MeSH Terms] OR ("allied"[tw] AND "health"[tw] AND "personnel"[tw]) OR "allied health personnel"[tw] OR "paramedics"[tw]) OR ("pharmacists"[MeSH Terms] OR "pharmacists"[tw] OR "pharmacist"[tw]) OR ("pharmacists"[MeSH Terms] OR "pharmacists"[tw] OR medic[tw] OR medics[tw])	1202459
#8	Search lab technician [tw] OR lab technicians [tw] OR laboratory technician [tw] OR laboratory technicians [tw]	1266
#10	Search medical resident[tw] OR medical residents[tw] OR medical graduates[tw] OR medical student[tw] OR medical students[tw]	46762
#7	Search "health services needs and demand"[MeSH Terms]	53722
#6	Search "health manpower"[MeSH Terms]	11792
#5	Search "health personnel"[MeSH Terms]	429668

#4	Search health worker [tw] OR health workers [tw] OR healthcare worker [tw] OR healthcare workers [tw] OR medical worker [tw] OR medical workers [tw] OR health professional [tw] OR health professionals [tw] OR healthcare professional [tw] OR healthcare professionals [tw] OR medical professional [tw] OR medical professionals [tw] OR health personnel [tw] OR healthcare personnel [tw] OR medical personnel [tw] OR health manpower [tw] OR healthcare manpower [tw] OR medical manpower [tw] OR health workforce [tw] OR healthcare workforce [tw] or medical workforce [tw]	236903
#3	Search human resource [tw] OR human resources [tw]	10366

CENTRAL

Search Name: **Bahrain**

Last Saved: 02/02/2017 10:56:37.043

Description:

ID Search

#1 worker or professional or personnel or manpower or workforce:ti,ab,kw and health or healthcare or medical:ti,ab,kw (Word variations have been searched)

#2 doctor or nurse or nursing or physician or midwife or midwives or midwifery or paramedic or medic or medics or pharmacist:ti,ab,kw (Word variations have been searched)

#3 birth:ti,ab,kw and "attendant":ti,ab,kw (Word variations have been searched)

#4 lab or laboratory:ti,ab,kw and "technician":ti,ab,kw (Word variations have been searched)

#5 medical:ti,ab,kw and resident or graduate or student:ti,ab,kw (Word variations have been searched)

#6 human:ti,ab,kw and "resource":ti,ab,kw (Word variations have been searched)

#7 #1 or #2 or #3 or #4 or #5 or #6

#8 "Bahrain":ti,ab,kw or "Bahraini":ti,ab,kw or bahrainis:ti,ab,kw (Word variations have been searched)

#9 #7 and #8

Search Name: **Egypt**

Last Saved: 02/02/2017 10:22:34.963

Description:

ID Search

#1 "Egypt":ti,ab,kw or "Egyptian":ti,ab,kw or egyptians:ti,ab,kw (Word variations have been searched)

#2 worker or professional or personnel or manpower or workforce:ti,ab,kw and health or healthcare or medical:ti,ab,kw (Word variations have been searched)

#3 doctor or nurse or nursing or physician or midwife or midwives or midwifery or paramedic or medic or medics or pharmacist:ti,ab,kw (Word variations have been searched)

#4 birth:ti,ab,kw and "attendant":ti,ab,kw (Word variations have been searched)

#5 lab or laboratory:ti,ab,kw and "technician":ti,ab,kw (Word variations have been searched)

#6 medical:ti,ab,kw and resident or graduate or student:ti,ab,kw (Word variations have been searched)

#7 human:ti,ab,kw and "resource":ti,ab,kw (Word variations have been searched)

#8 #2 or #3 or #4 or #5 or #6 or #7

#9 #1 and #8 Publication Year from 2011 to 2017, in Cochrane Reviews (Reviews and Protocols), Other Reviews, Trials, Technology Assessments and Economic Evaluations

Search Name: **Syria**

Last Saved: 02/02/2017 11:23:47.432

Description:

ID Search

#1 worker or professional or personnel or manpower or workforce:ti,ab,kw and health or healthcare or medical:ti,ab,kw (Word variations have been searched)

#2 doctor or nurse or nursing or physician or midwife or midwives or midwifery or paramedic or medic or medics or pharmacist:ti,ab,kw (Word variations have been searched)

#3 birth:ti,ab,kw and "attendant":ti,ab,kw (Word variations have been searched)

#4 lab or laboratory:ti,ab,kw and "technician":ti,ab,kw (Word variations have been searched)

#5 medical:ti,ab,kw and resident or graduate or student:ti,ab,kw (Word variations have been searched)

#6 human:ti,ab,kw and "resource":ti,ab,kw (Word variations have been searched)

#7 #1 or #2 or #3 or #4 or #5 or #6

#8 "Syria":ti,ab,kw or "Syrian":ti,ab,kw or syrians:ti,ab,kw (Word variations have been searched)

#9 #7 and #8 Publication Year from 2011 to 2017, in Cochrane Reviews (Reviews and Protocols), Other Reviews, Trials, Technology Assessments and Economic Evaluations

Search Name: **Iraq**

Last Saved: 02/02/2017 11:06:10.052

Description:

ID Search

#1 worker or professional or personnel or manpower or workforce:ti,ab,kw and health or healthcare or medical:ti,ab,kw (Word variations have been searched)

#2 doctor or nurse or nursing or physician or midwife or midwives or midwifery or paramedic or medic or medics or pharmacist:ti,ab,kw (Word variations have been searched)

#3 birth:ti,ab,kw and "attendant":ti,ab,kw (Word variations have been searched)

#4 lab or laboratory:ti,ab,kw and "technician":ti,ab,kw (Word variations have been searched)

#5 medical:ti,ab,kw and resident or graduate or student:ti,ab,kw (Word variations have been searched)

#6 human:ti,ab,kw and "resource":ti,ab,kw (Word variations have been searched)

#7 #1 or #2 or #3 or #4 or #5 or #6

#8 "Iraq":ti,ab,kw or "Iraqi":ti,ab,kw or Iraqis:ti,ab,kw or "Iraqian":ti,ab,kw or iraqians (Word variations have been searched)

#9 #7 and #8 Publication Year from 2003 to 2017, in Cochrane Reviews (Reviews and Protocols), Other Reviews, Trials, Technology Assessments and Economic Evaluations

Search Name: **Yemen**

Last Saved: 06/02/2017 09:45:52.526

Description:

ID Search

#1 worker or professional or personnel or manpower or workforce:ti,ab,kw and health or healthcare or medical:ti,ab,kw (Word variations have been searched)

#2 doctor or nurse or nursing or physician or midwife or midwives or midwifery or paramedic or medic or medics or pharmacist:ti,ab,kw (Word variations have been searched)

#3 birth:ti,ab,kw and "attendant":ti,ab,kw (Word variations have been searched)

#4 lab or laboratory:ti,ab,kw and "technician":ti,ab,kw (Word variations have been searched)

#5 medical:ti,ab,kw and resident or graduate or student:ti,ab,kw (Word variations have been searched)

#6 human:ti,ab,kw and "resource":ti,ab,kw (Word variations have been searched)

#7 #1 or #2 or #3 or #4 or #5 or #6

#8 "Yemen":ti,ab,kw or "Yemen Arab Republic":ti,ab,kw or "Yemeni":ti,ab,kw or yemenis:ti,ab,kw (Word variations have been searched)

#9 #7 and #8 Publication Year from 2011 to 2017

Search Name: **Libya**

Last Saved: 02/02/2017 12:54:24.423

Description:

ID Search

#1 worker or professional or personnel or manpower or workforce:ti,ab,kw and health or healthcare or medical:ti,ab,kw (Word variations have been searched)

#2 doctor or nurse or nursing or physician or midwife or midwives or midwifery or paramedic or medic or medics or pharmacist:ti,ab,kw (Word variations have been searched)

#3 birth:ti,ab,kw and "attendant":ti,ab,kw (Word variations have been searched)

#4 lab or laboratory:ti,ab,kw and "technician":ti,ab,kw (Word variations have been searched)

#5 medical:ti,ab,kw and resident or graduate or student:ti,ab,kw (Word variations have been searched)

#6 human:ti,ab,kw and "resource":ti,ab,kw (Word variations have been searched)

#7 #1 or #2 or #3 or #4 or #5 or #6

#8 "Libya":ti,ab,kw or "Libyan":ti,ab,kw or libyans:ti,ab,kw (Word variations have been searched)

#9 #7 and #8 Publication Year from 2011 to 2017, in Cochrane Reviews (Reviews and Protocols), Other Reviews, Trials, Technology Assessments and Economic Evaluations.

Search Name: **Tunisia**

Last Saved: 02/02/2017 11:12:12.484

Description:

ID Search

#1 worker or professional or personnel or manpower or workforce:ti,ab,kw and health or healthcare or medical:ti,ab,kw (Word variations have been searched)

#2 doctor or nurse or nursing or physician or midwife or midwives or midwifery or paramedic or medic or medics or pharmacist:ti,ab,kw (Word variations have been searched)

#3 birth:ti,ab,kw and "attendant":ti,ab,kw (Word variations have been searched)

#4 lab or laboratory:ti,ab,kw and "technician":ti,ab,kw (Word variations have been searched)

#5 medical:ti,ab,kw and resident or graduate or student:ti,ab,kw (Word variations have been searched)

#6 human:ti,ab,kw and "resource":ti,ab,kw (Word variations have been searched)

#7 #1 or #2 or #3 or #4 or #5 or #6

#8 "Tunisia":ti,ab,kw or "Tunisian":ti,ab,kw or Tunisians:ti,ab,kw (Word variations have been searched)

#9 #7 and #8 Publication Year from 2011 to 2017, in Cochrane Reviews (Reviews and Protocols), Other Reviews, Trials, Technology Assessments and Economic Evaluations

WHO Global Health Library

Syria: 280

Syria* AND (instance:"ghl") AND (fulltext:("1") AND mj:("Refugees" OR "War" OR "Delivery of Health Care" OR "Syria" OR "Physicians" OR "Health Services Accessibility" OR "Public Health" OR "Arabs" OR "International Cooperation" OR "Wounds, Gunshot" OR "Human Rights Abuses" OR "Health Services Needs and Demand" OR "Violence" OR "Wounds and Injuries" OR "Health Services" OR "Islam" OR "Politics" OR "Health Status" OR "Terrorism" OR "Egypt" OR "Physician's Role" OR "Stress, Psychological" OR "Charities" OR "Civil Disorders" OR "Health Personnel" OR "Developing Countries" OR "Health Policy" OR "Students, Medical" OR "Emigration and Immigration" OR "Emigrants and Immigrants" OR "Maternal Health Services" OR "Medicine, Arabic" OR "Mental Disorders" OR "Primary Health Care" OR "Quality of Health Care" OR "Sarin" OR "Schools, Medical" OR "Stress Disorders, Post-Traumatic" OR "Transients and Migrants" OR "Universities" OR "Ethnic Groups" OR "Mental Health" OR "Health Surveys" OR "Cause of Death" OR "Health Planning" OR "Hospitals" OR "Mental Health Services" OR "Organization and Administration"))

AND (instance:"ghl") AND (instance:"ghl") AND (year_cluster:("2015" OR "2013" OR "2014" OR "2016" OR "2012" OR "2011"))

Iraq: 2248

Iraq* AND (instance:"ghl") AND (fulltext:("1") AND mj:("Military Personnel" OR "Iraq War, 2003-2011" OR "Stress Disorders, Post-Traumatic" OR "Veterans" OR "War" OR "Wounds and Injuries" OR "Military Medicine" OR "Brain Injuries" OR "Combat Disorders") AND year_cluster:("2011" OR "2010" OR "2008" OR "2009" OR "2003" OR "2005" OR "2007" OR "2004" OR "2006" OR "2012" OR "2013" OR "2014" OR "2015" OR "2016"))

Bahrain: 150

bahrain* AND (instance:"ghl") AND (year_cluster:("2011" OR "2012" OR "2015"))

Libya: 402

Libya* AND (instance:"ghl") AND (fulltext:("1") AND year_cluster:("2014" OR "2012" OR "2015" OR "2013" OR "2011" OR "2016"))

Yemen: 505

Yemen* AND (instance:"ghl") AND (fulltext:("1") AND year_cluster:("2014" OR "2015" OR "2012" OR "2013" OR "2016" OR "2011"))

Egypt: 109

Egypt* AND (instance:"ghl") AND (fulltext:("1") AND mj:("Health Knowledge, Attitudes, Practice" OR "Rural Health Services" OR "Islam" OR "Pharmacy Service, Hospital" OR "Prenatal Care" OR "Primary Health Care" OR "Students" OR "Students, Medical" OR "Healthcare Disparities" OR "Family Practice" OR "Public Health Surveillance" OR "Health Promotion" OR "Hospitalization" OR "Hospitals" OR "Maternal Health Services" OR "Nursing Staff, Hospital") AND year_cluster:("2014" OR "2013" OR "2015" OR "2012"))

Tunisia: 14

Tunisia* AND (instance:"ghl") AND (fulltext:("1") AND mj:("Arabs") AND year_cluster:("2012" OR "2013" OR "2014" OR "2015" OR "2011"))

Appendix S2: List of included studies

1. Abbara A, Orcutt M, Gabbar O. Syria's lost generation of doctors. Bmj. 2015;350:h3479. doi:10.1136/bmj.h3479
2. AbuAlRub RF, Khalifa MF, Habbib MB. Workplace violence among Iraqi hospital nurses. J Nurs Scholarsh. 2007;39(3):281-288. doi:10.1111/j.1547-5069.2007.00181.x
3. Ahmed F. Doctors in Syria: saving the lifesavers. Lancet. 2013; 382(9905):1619. doi:10.1016/s0140-6736(13)62248-5
4. Ahsan S. Providing medical relief in Syria's conflict. Lancet 2013; 381: 523–24.
5. Al Mosawi AJ. Medical education and the physician workforce of Iraq. J Contin Educ Health Prof. 2008;28(2):103-105. doi:10.1002/chp.166
6. Al Sheibani BI, Hadi NR, Hasoon T. Iraq lacks facilities and expertise in emergency medicine. Bmj. 2006;333(7573):847. doi:10.1136/bmj.38986.476782.68
7. Ali Jadoo SA, Aljunid SM, Dastan I, et al. Job satisfaction and turnover intention among Iraqi doctors-a descriptive cross-sectional multicentre study. Hum Resour Health. 2015;13:21. doi:10.1186/s12960-015-0014-6
8. Al-Khaled M. Syrian public health and doctors. Lancet. 2012;380(9844):804. doi:10.1016/s0140-6736(12)61448-2
9. Al-Khalisi N. The Iraqi medical brain drain: a cross-sectional study. Int J Health Serv. 2013;43(2):363-378.
10. Al-Khalisi N. The perils of being a doctor in Baghdad. Bmj. 2010;341:c4043. doi:10.1136/bmj.c4043
11. Al-Kindi S. Violence against doctors in Iraq. Lancet. 2014;384(9947):954-955. doi:10.1016/s0140-6736(14)61627-5
12. Alwan NA. The killing of doctors in Iraq must stop. Bmj. 2011;343:d4467. doi:10.1136/bmj.d4467
13. Amin NM, Khoshnaw MQ. Medical education and training in Iraq. Lancet. 2003;362(9392):1326.
14. Torture in Syria's hospitals. Lancet. 2011;378(9803):1606-1606.
15. A medical crisis in Syria. Lancet. 2012;380(9841):537. doi:10.1016/s0140-6736(12)61309-9
16. Syria: a health crisis too great to ignore. Lancet. 2016;388(10039):2. doi:10.1016/s0140-6736(16)30936-9
17. Bahraini hospital recruitment drive continues despite unrest. Nurs Stand. 2011;25(37):11. doi:10.7748/ns.25.37.11.s18
18. Anonymous. Letter of support for Egyptian health workers. Nursing Standard. 2011;25(23):6.
19. Anonymous. Convicted nurse calls on global community to act. Nursing Standard. 2012;26(31):8.
20. Charities send aid and staff to Libya. Nursing Standard. 2011;26(1):6. doi:10.7748/ns.26.1.6.s5
21. Vote to help workforce in Bahrain. Nurs Stand. 2011;25(36):11. doi:10.7748/ns.25.36.11.s18

22. Anonymous. Bahrain nurse leader jailed 15 years for 'ludicrous charges'. Nursing Standard. 2011;26(5):8.
23. Anonymous. Syrian woman on the way towards licensure as pharmacist: Chamber of Westphalia-Lippe accepted first terminology examination. Deutsche Apotheker Zeitung. 2016;156(36).
24. Anonymous. At last working in "dreamland": Syrian pharmacist is now working as "pharmacist under supervision". Deutsche Apotheker Zeitung. 2016;156 (7).
25. Arie S. Bahraini doctors are promised civilian trials after protests over harsh sentences. Bmj. 2011;343:d6475. doi:10.1136/bmj.d6475
26. Arie S. Bahraini doctors' salaries are axed as they await retrial for crimes against the state. Bmj. 2011;343:d7071. doi:10.1136/bmj.d7071
27. Arie S. Bahraini doctors deny anti-state activities. Bmj. 2011;342:d3755. doi:10.1136/bmj.d3755
28. Arie S. Bahraini doctors start hunger strike over arrests and trials in military court. Bmj. 2011;343:d5673. doi:10.1136/bmj.d5673
29. Arie S. Commission finds no evidence that Bahraini doctors refused to treat Sunni patients, but appeal continues. Bmj. 2011;343:d7800. doi:10.1136/bmj.d7800
30. Arie S. Doctors who treated protesters in Bahrain are given 15 year sentences. Bmj. 2011;343:d6336. doi:10.1136/bmj.d6336
31. Arie S. Syrian doctors risk arrest and deportation for treating fellow refugees in Lebanon and Jordan. Bmj. 2015;350:h1552. doi:10.1136/bmj.h1552
32. Arie S. Irish surgeons college admits it treated arrested doctors “insensitively”. BMJ. 2011;343:d6719-d6719.
33. Arie S. Some charges against Bahraini doctors are dropped, but more serious crimes are added. Bmj. 2011;343:d6910. doi:10.1136/bmj.d6910
34. Arie S. Gaddafi's forces attacked hospitals, patients, and health professionals, report confirms. Bmj. 2011;343:d5533. doi:10.1136/bmj.d5533
35. Arie S. Twenty one Bahraini medical professionals overturn convictions for anti-government protest. Bmj. 2013;346:f2104. doi:10.1136/bmj.f2104
36. Arie S. Syrian doctor who gave medical aid to protesters dies in custody. Bmj. 2013;347:f5951. doi:10.1136/bmj.f5951
37. Arie S. Nine Bahraini medical professionals are sentenced to five years in prison. Bmj. 2012;344:e4216. doi:10.1136/bmj.e4216
38. Arie S, Houston M. Promise of retrial for Bahraini doctors was for Western eyes only. BMJ. 2011;343:d6567. doi:10.1136/bmj.d6567
39. Arie, S. Medical staff detained in Bahrain are released on bail after hunger strike. BMJ 2011;343 doi:http://dx.doi.org/10.1136/bmj.d5749
40. Arie, S. Bahraini authorities jail 23 medical staff for three months for role in protests. BMJ 2012, 345 doi:http://dx.doi.org/10.1136/bmj.e8004
41. Arie S. Medical groups press Bahrain over detention of doctors BMJ 2011; 343 :d4248
42. Attar S. Field hospitals in Syria. Lancet. 2014;383(9914):303-303.

43. Barnett-Vanes A, Hassounah S, Shawki M, et al. Impact of conflict on medical education: a cross-sectional survey of students and institutions in Iraq. BMJ Open. 2016;6(2):e010460. doi:10.1136/bmjopen-2015-010460
44. Ben Taleb Z, Bahelah R, Fouad FM, Coutts A, Wilcox M, Maziak W. Syria: health in a country undergoing tragic transition. Int J Public Health. 2015;60 Suppl 1:S63-72. doi:10.1007/s00038-014-0586-2
45. Brundtland GH, Glinka E, Hausen HZ, D'Avila RL. Open letter: Let us treat patients in Syria. The Lancet. 2013;382(9897):1019-1020.
46. Burnham GM, Lafta R, Doocy S. Doctors leaving 12 tertiary hospitals in Iraq, 2004-2007. Soc Sci Med. 2009;69(2):172-177. doi:10.1016/j.socscimed.2009.05.021
47. Burnham G, Malik S, Al-Shibli AS, et al. Understanding the impact of conflict on health services in Iraq: information from 401 Iraqi refugee doctors in Jordan. Int J Health Plann Manage. 2012;27(1):e51-64. doi:10.1002/hpm.1091
48. Cousins S. Iraq: staff and medicine shortages are major challenges. Lancet. 2014;384(9947):943-944.
49. Cousins S. Syrian crisis: health experts say more can be done. Lancet. 2015;385(9972):931-934.
50. Cousins S. Under attack: Aleppo's hospitals. Lancet. 2014;384(9939):221-222.
51. Coutts A, McKee M, Stuckler D. The emerging Syrian health crisis. Lancet. 2013;381(9865):e6-7.
52. De Leeuw M. Syrian pharmacist was in danger and fled to the Netherlands: Help the injured. Pharmaceutisch Weekblad. 2014;149(51-52):66.
53. Devi S. Syria's health crisis: 5 years on. Lancet. 2016;387(10023):1042-1043. doi:10.1016/s0140-6736(16)00690-5
54. Devi S. Health professionals under threat in Bahrain. Lancet. 2011;377(9779):1733-1734.
55. Devi S. Medical community urged to defend Bahraini doctors. Lancet. 2011;378(9799):1287.
56. Devi S. Bahrain continues to target Shia doctors. Lancet. 2012;380(9850):1296.
57. Devi S. Bahrain convicts more health professionals. Lancet. 2012;380(9857):1895.
58. Devi S. Bahrain reduces sentences for protest doctors and nurses. Lancet. 2012;379(9834):2327.
59. Ditzel P. "Germany is like a dreamland": How a Syrian pharmacist wants to settle down in Germany. Deutsche Apotheker Zeitung. 2015;155(38).
60. Donaldson RI, Hasson T, Aziz S, Ansari W, Evans G. The development of civilian emergency medical care during an insurgency: current status and future outlook in Iraq. Ann Emerg Med. 2010;56(2):172-177. doi:10.1016/j.annemergmed.2009.12.028
61. Donaldson RI, Mulligan DA, Nugent K, et al. Using tele-education to train civilian physicians in an area of active conflict: certifying Iraqi physicians in Pediatric Advanced Life Support from the United States. J Pediatr. 2011;159(3):507-509.e501. doi:10.1016/j.jpeds.2011.05.003
62. Donaldson RI, Shanovich P, Shetty P, et al. A survey of national physicians working in an active conflict zone: the challenges of emergency medical care in Iraq. Prehosp Disaster Med. 2012;27(2):153-161. doi:10.1017/s1049023x12000519

63. Doocy S, Malik S, Burnham G. Experiences of Iraqi doctors in Jordan during conflict and factors associated with migration. Am J Disaster Med. 2010;5(1):41-47.
64. Dyer O. Doctors forced to guard Baghdad hospitals after looting. Bmj. 2003;326(7394):837. doi:10.1136/bmj.326.7394.837
65. Dyer O. Baghdad’s hospitals struggle to cope with war wounded. BMJ. 2003;326(7393):779-779.
66. Dyer O. Iraqi healthcare system still crippled five years after invasion, says report. BMJ. 2008;336(7636):113-113.
67. Dyer C. Bahraini doctor detained for Tweets needs legal and medical support, says BMA. Bmj. 2016;352:i300. doi:10.1136/bmj.i300
68. Fernandez G, Boulle P. Where conflict's medical consequences remain unchanged. Lancet. 2013;381(9870):901. doi:10.1016/s0140-6736(13)60664-9
69. Fleck K. Medical care in Syria: Doctors in a hail of bombs. Deutsches Arzteblatt International. 2015;112(51-52):A2194-A2195.
70. Fouad FM, Alameddine M, Coutts A. Human resources in protracted crises: Syrian medical workers. Lancet. 2016;387(10028):1613. doi:10.1016/s0140-6736(16)30197-0
71. Friedrich MJ. Human rights report details violence against health care workers in Bahrain. Jama. 2011;306(5):475-476. doi:10.1001/jama.2011.1091
72. Friedrich MJ. Bahrain continues aggressive action against its health care system. Jama. 2012;308(7):655-656. doi:10.1001/jama.2012.9673
73. Furber AS, Johnstone P. Rebuilding health care in Iraq. J Epidemiol Community Health. 2004;58(11):890-892.
74. Gardham D. Time for tighter checks on medical schools? BMJ. 2015;350:h3511-h3511.
75. Garfield R, McCarthy CF. Nursing and nursing education in Iraq: challenges and opportunities. Int Nurs Rev. 2005;52(3):180-185. doi:10.1111/j.1466-7657.2005.00428.x
76. Garfield R. Health professionals in Syria. *Lancet.* 2013;382(9888):205-206. doi:10.1016/s0140-6736(13)61507-x
77. Gavin J. Syrian government defies principle of medical neutrality by targeting underground clinics. BMJ. 2011;343:d8314-d8314.
78. Ghaleb S, Mukwege DM, Roberts R, Sulkowicz KJ, Vlassov VV, signatories aflosiaita. Protect Syria's doctors: an open letter to world leaders. Lancet. 2016;388(10049):1056.
79. Godlee F. Editor's choice: Support Bahrain's imprisoned doctors. BMJ (Online). 2011;343 (7826) (no pagination)(d6417).
80. Gray B, Ockelford P. Bahrain health workers in danger: call to action. N Z Med J. 2011;124(1344):109-110.
81. Gulland A. Volunteer doctors are "better organised" to treat casualties in Tahrir Square than during previous unrest. Bmj. 2011;343:d7666. doi:10.1136/bmj.d7666
82. Gulland A. WHO condemns attacks on healthcare workers in Yemen. Bmj. 2015;350:h2914. doi:10.1136/bmj.h2914
83. Gulland A. Doctors urge Syrian government to allow them access to patients. Bmj. 2013;347:f5698. doi:10.1136/bmj.f5698
84. Gulland A. Medical students perform operations in Syria's depleted health system. Bmj. 2013;346:f3107. doi:10.1136/bmj.f3107

85. Gulland A. Doctors in Syria are being forced to treat patients in secret, charity says. Bmj. 2012;344:e1000. doi:10.1136/bmj.e1000
86. Gulland A. Syrian doctors tell charity that: “being caught with patients is like being caught with a weapon”. BMJ. 2012;344:e3506-e3506.
87. Haidar S, Lehr M. When Syrian pharmacists want to work here: Is the training for pharmacists in Syria comparable with that in Germany? Deutsche Apotheker Zeitung. 2016;156(32):147.
88. Hallam R. Response to Syria’s health crisis. Lancet. 2013;382(9893):679-680.
89. Hampton T. Health care under attack in Syrian conflict. Jama. 2013;310(5):465-466. doi:10.1001/jama.2013.69374
90. Hathout L. The right to practice medicine without repercussions: ethical issues in times of political strife. Philos Ethics Humanit Med. 2012;7:11. doi:10.1186/1747-5341-7-11
91. Heisler M, Baker E, McKay D. Attacks on health care in Syria - Normalizing violations of medical neutrality? New England Journal of Medicine. 2015;373(26):2489-2491.
92. Iacobucci G. Syrian doctors appeal to President Obama for help. Bmj. 2016;354:i4458. doi:10.1136/bmj.i4458
93. Jackson G. Stop: the medical and human rights scandal in Bahrain. Int J Clin Pract. 2011;65(8):823. doi:10.1111/j.1742-1241.2011.02736.x
94. Johna S. The anatomy of crime: physicians under the oppressive regime of Saddam Hussein. Acta Med Hist Adriat. 2009;7(2):303-308.
95. Kapp C. Anarchy pushes Iraqi health system to brink of collapse: Doctors arm themselves to protect their hospitals from looters. Lancet. 2003;361(9366):1351.
96. Kassem M. A Syrian neurosurgeon's journey. Surgical Neurology International. 2016;7 (1) (no pagination)(6).
97. Kevat D, Lander F, Loff B. The growing threat to medical independence in conflict zones. The Lancet. 2012;379(9814):e31-e32.
98. Lazarus C. Imprisonment and torture of doctors in Bahrain. S Afr Med J. 2012;102(6 Pt 2):336.
99. Martin A, Post N, Martin M. Syria: What should health care professionals do? Journal of Global Health. 2014;4(1):010302.
100. Mason P, Ghareeb K. Iraqi pharmacists contend with violence, murder and uncertainty... but in northern Iraq the future looks brighter. Pharmaceutical Journal. 2007;279(7480):624
101. McCarthy C. Helping Iraqi nurses get back on track. Interview by Miriam McCauley. Nursing. 2004;34(2):44-45.
102. Michaud EC, 3rd, Maxwell GL. Medical capacity building efforts in northern Iraq 2009-2010. Mil Med. 2012;177(6):676-680.
103. Mills S, Arie S. Irish surgeons' college admits it questioned Bahraini medical students after being contacted by government. Bmj. 2011;343:d6616. doi:10.1136/bmj.d6616
104. Nathanson V, Chrispin E. Bahraini health workers to be retried before a civilian court. BMJ. 2011;343:d6547. doi:10.1136/bmj.d6547
105. Neville P. Iraqi nurses too scared to work. Nurs Stand. 2003;17(32):7. doi:10.7748/ns.17.32.7.s14

106. O'Brien E. Bahrain: continuing imprisonment of doctors. Lancet. 2011;378(9798):1203-1205. doi:10.1016/s0140-6736(11)61353-6
107. Patterson V, Swinfen P, Swinfen R, Azzo E, Taha H, Wootton R. Supporting hospital doctors in the Middle East by email telemedicine: something the industrialized world can do to help. J Med Internet Res. 2007;9(4):e30. doi:10.2196/jmir.9.4.e30
108. Pincock S. Salam Ismael. Lancet. 2006;368(9533):359. doi:10.1016/s0140-6736(06)69094-6
109. Rawaf S, Dubois E, Kubba A, et al. Iraqi doctors' training in UK: Looking to rebuild Iraq's healthcare system. BMJ (Online). 2009;339(7730):1101.
110. Reis C, Ahmed AT, Amowitz LL, Kushner AL, Elahi M, Iacopino V. Physician participation in human rights abuses in southern Iraq. Jama. 2004;291(12):1480-1486. doi:10.1001/jama.291.12.1480
111. Reiss-Koncar D. Bandaging Baghdad. Revolution. 2003;4(4):18-25.
112. Salvage J. Against all the odds. Nurs Stand. 2003;17(49):14-15.
113. Salvage J. Nurses are key, even here in Iraq. Nurs Times. 2004;100(5):16.
114. Sankari A, Atassi B, Sahloul MZ. Syrian field hospitals: A creative solution in urban military conflict combat in Syria. Avicenna J Med. 2013;3(3):84-86. doi:10.4103/2231-0770.118467
115. Saunders S, London L. Health professionals should be speaking out about the victimization of doctors in Bahrain. South African Medical Journal. 2012;102(3):113-114.
116. Sekkarie MA, Murad L, Sahloul Z. Revival of basic health services in Syria. Lancet Glob Health. 2015;3(10):e597-e597.
117. Shukla S. Violence against doctors in Egypt leads to strike action. Lancet. 2012;380(9852):1460.
118. Sibbald B. Syria mission: safe is a relative term for Ottawa doctor. Cmaj. 2013;185(10):E453-454. doi:10.1503/cmaj.109-4504
119. Sibbald B. Physicians, health facilities targeted in war-torn Syria. Cmaj. 2013;185(9):755-756. doi:10.1503/cmaj.109-4492
120. Slim A. "Over the shoulder" training: impact on the experience of local iraqi physicians. J Grad Med Educ. 2012;4(1):117-118. doi:10.4300/jgme-d-11-00198.1
121. Sollom R, Iacopino V. Bahrain unrest. BMJ. 2011;342:d2768-d2768.
122. Sonderup M. SAMA speaks out about the victimisation of doctors in Bahrain. S Afr Med J. 2012;102(6 Pt 2):336.
123. Squires A, Sindi A, Fennie K. Reconstructing a health system and a profession: priorities of Iraqi nurses in the Kurdish region. ANS Adv Nurs Sci. 2006;29(1):55-68.
124. Squires A, Sindi A, Fennie K. Health system reconstruction: Perspectives of Iraqi physicians. Glob Public Health. 2010;5(6):561-577. doi:10.1080/17441690903473246
125. Stone-Brown K. Syria: a healthcare system on the brink of collapse. Bmj. 2013;347:f7375. doi:10.1136/bmj.f7375
126. Van Den Heuvel M. (No) a strategy against the shortage of skilled labor? Syrian pharmacists in Germany. Deutsche Apotheker Zeitung. 2016;156(21).

127. Voelker R. Iraq's prescription for violent barriers to health care: Cell phones and e-mail. JAMA - Journal of the American Medical Association. 2008;300(6):637-638.
128. Vogel L. Why are doctors joining ISIS? Cmaj. 2016;188(3):177-178. doi:10.1503/cmaj.109-5217
129. Webster P. Medical faculties decimated by violence in Iraq. Cmaj. 2009;181(9):576-578. doi:10.1503/cmaj.109-3035
130. Webster P. Reconstruction efforts in Iraq failing health care. Lancet. 2009;373(9664):617-620.
131. Webster PC. Iraq's health system yet to heal from ravages of war. Lancet. 2011;378(9794):863-866.
132. Webster P. Facility attacks in Syria contravene Geneva Convention. CMAJ. 2016;188(7):491-491.
133. Zarocostas J. Exodus of medical staff strains Iraq's health facilities. Bmj. 2007;334(7599):865. doi:10.1136/bmj.39195.466713.DB
134. Zarocostas J. Hospitals in Syria have become instruments of suppression, says Amnesty. BMJ. 2011;343:d6947-d6947.
135. Zarocostas J. Libyan health system is “absolutely stretched” says UN. BMJ. 2011;343:d4326-d4326.
136. Zarocostas, J. Human rights group calls on bahrain to release medical personnel and remove security forces from hospitals. BMJ 2011, 343 doi:http://dx.doi.org/10.1136/bmj.d4781
